# Poster Session I - A151 PILOT VIRTUAL EDUCATIONAL WEBSITE FOR PEDIATRIC DISORDERS OF GUT-BRAIN INTERACTION

**DOI:** 10.1093/jcag/gwaf042.151

**Published:** 2026-02-13

**Authors:** B Chen, M W Carroll, D M Isaac, J Silverman, J Turner

**Affiliations:** Pediatric gastroenterology, Stollery Children’s Hospital, Edmonton, AB, Canada; Pediatric gastroenterology, Stollery Children’s Hospital, Edmonton, AB, Canada; Pediatric gastroenterology, Stollery Children’s Hospital, Edmonton, AB, Canada; Pediatric gastroenterology, Stollery Children’s Hospital, Edmonton, AB, Canada; Pediatric gastroenterology, Stollery Children’s Hospital, Edmonton, AB, Canada

## Abstract

**Background:**

Disorders of gut-brain interaction (DGBIs) are common conditions affecting more than a quarter of children globally. Patients with these disorders have reduced quality of life and suffer from increased anxiety, depression, sleep problems and school absenteeism. DGBIs impact the entire family and management of these disorders is costly and resource-intensive. Minimal education is provided on DGBIs during medical training. While many primary healthcare providers may feel ill-equipped to diagnose and manage these disorders, children with DGBIs can be safely managed in the community without the need for gastroenterology referral and expensive tertiary level investigations.

**Aims:**

We aimed to develop an online resource to deliver education on common pediatric DGBIs to provide community physicians with knowledge to confidently diagnose and manage these conditions. We also developed a resource section for providers, patients and families.

**Methods:**

We designed an online educational program on pediatric DGBIs comprising of eight on-demand modules with presentations recorded by pediatric gastroenterologists at the Stollery Children’s Hospital. The website was launched on July 1, 2023 and advertised to local pediatricians, family physicians and learners. Users completed pre- and post-module knowledge questions to measure module impact. Feedback evaluations were completed for module quality assessment and improvement. Participants rated the perceived overall impact of each training video on a Likert scale from 1-5, with 5 as most positive. Additionally, we developed a resources section with links and handouts for patients and families.

**Results:**

Between July 1, 2023 to September 1, 2025, over 100 individuals have registered. However, module completion rates were low between 2 to 13 users per module. Across all modules, 135 pre- and post- questions were completed. In total, the proportion of correct answers increased from 53% to 66% (Pearson Chi-Square, p < 0.001). Baseline knowledge was variable according to individual module, with the proportion of correct answers ranging from 26-73%. Overall feedback was positive (average score 4/5, range 2.3-4.7) across all modules. Majority of users indicated they would recommend the program to colleagues. Since tracking website traffic in February 2025, there have been 136 visitors (62% clinicians, 35% families/patients, 3% other) to the website resource page.

**Conclusions:**

Knowledge of common DGBI impacting children is low for community health care providers. While an online education program appear to enhance absolute increase in knowledge, it can be quite variable according to topic. This approach may be limited by low rate of module completion after program registration. Despite this, initial feedback has been positive, suggesting this approach is in part addressing a gap or concern for providers.

**Funding Agencies:**

None

